# Association of anti‐calcitonin gene‐related peptide with other monoclonal antibodies for different diseases: A multicenter, prospective, cohort study

**DOI:** 10.1111/ene.16450

**Published:** 2024-09-16

**Authors:** Luigi Francesco Iannone, Marina Romozzi, Antonio Russo, Gennaro Saporito, Federico De Santis, Raffaele Ornello, Grazia Sances, Gloria Vaghi, Cristina Tassorelli, Maria Albanese, Simona Guerzoni, Alfonsina Casalena, Catello Vollono, Paolo Calabresi, Maria Pia Prudenzano, Edoardo Mampreso, Giorgio Dalla Volta, Maria Rosaria Valente, Gianluca Avino, Alberto Chiarugi, Simona Sacco, Francesca Pistoia, the Italian Headache Registry (RICe) study group, Francesca Boscain, Roberta Bovenzi, Marco Bolchini, Matteo Cortinovis, Roberto De Icco, Natascia Ghiotto, Flavia Lo Castro, Andrea Burgalassi, Giulia Vigani, Francesco De Cesaris, Nicola Biagio Mercuri, Viviana Nociti

**Affiliations:** ^1^ Section of Clinical Pharmacology and Oncology, Department of Health Sciences University of Florence Florence Italy; ^2^ Fondazione Policlinico Universitario Agostino Gemelli IRCCS Università Cattolica del Sacro Cuore Rome Italy; ^3^ Headache Center, Department of Advanced Medical and Surgical Sciences University of Campania “Luigi Vanvitelli” Naples Italy; ^4^ Department of Biotechnological and Applied Clinical Sciences University of L'Aquila L'Aquila Italy; ^5^ Headache Science and Neurorehabilitation Unit IRCCS Mondino Foundation Pavia Italy; ^6^ Department of Brain and Behavioral Sciences University of Pavia Pavia Italy; ^7^ Regional Referral Headache Center, Neurology Unit Tor Vergata University Hospital Rome Italy; ^8^ Digital and Predictive Medicine, Pharmacology, and Clinical Metabolic Toxicology, Headache Center and Drug Abuse Laboratory of Clinical Pharmacology and Pharmacogenomics, Department of Specialist Medicines AOU Policlinico di Modena Modena Italy; ^9^ Neurology Unit “G. Mazzini” Hospital Teramo Italy; ^10^ Headache Center, Department of Basic Medical Sciences, Neurosciences, and Sense Organs University of Bari Bari Italy; ^11^ Headache Center, Neurology–Euganea Health Unit Padua Italy; ^12^ Headache Center of Clinical Neurology of Istituto Clinico Città di Brescia Brescia Italy; ^13^ Clinical Neurology Azienda Sanitaria Universitaria Friuli Centrale, Presidio Ospedaliero Santa Maria della Misericordia Udine Italy; ^14^ Neurology Unit, Ospedale Santo Stefano USL Toscana Centro Prato Italy; ^15^ Headache Center and Clinical Pharmacology Unit Careggi University Hospital Florence Italy

**Keywords:** CGRP, migraine, monoclonal antibodies, pharmacokinetic, pharmacological interactions

## Abstract

**Background and purpose:**

Although there is extensive evidence about the safety of monoclonal antibodies against calcitonin gene‐related peptide (anti‐CGRP mAbs) in combination with traditional drugs, scarce data are available on the safety of their combination with other mAbs. This study aimed to evaluate the 6‐month effectiveness and tolerability of anti‐CGRP mAbs in combination with other mAbs for different diseases.

**Methods:**

Patients included in the Italian Headache Registry and treated concomitantly with an anti‐CGRP mAb and another mAb were included. Effectiveness outcomes for migraine included reduction from baseline of monthly headache days (MHDs), Migraine Disability Assessment (MIDAS) score, Headache Impact Test‐6 (HIT‐6) scores, and Patients' Global Impression of Change (PGIC) scale. Adverse events (AEs) were recorded.

**Results:**

Thirty‐eight patients were included. In 27 patients (71.1%), the anti‐CGRP mAb was added to a previously ongoing mAb. Nine patients (23.7%) discontinued one of the two mAbs before the end of treatment (seven discontinued the anti‐CGRP mAb and two the other mAb). One patient discontinued for AEs. Anti‐CGRP mAbs were discontinued due to ineffectiveness (*n* = 5, 55.5%) and one each (11.1%) for clinical remission and lost to follow‐up. MHDs significantly decreased from baseline to 3 months (*p* < 0.0001) and 6 months (*p* < 0.001), as did the MIDAS and the HIT‐6 scores at 3 and 6 months (*p* < 0.001). For anti‐CGRP mAbs, 27.4% of patients reported PGIC ≥ 5 at 3 months and 48.3% at 6 months. Mild AEs associated with introduction of a second mAb were detected in six patients (15.8%).

**Conclusions:**

In this real‐world study, anti‐CGRP mAbs showed safety and effectiveness when administered concomitantly with other mAbs.

## INTRODUCTION

Monoclonal antibodies acting on the calcitonin gene‐related peptide (CGRP) pathway (anti‐CGRP mAbs) represent the first agents specifically designed for migraine prevention [1]. They are safe and effective therapies for patients who have failed several previous preventive strategies [2, 3]. Patients with migraine often have multiple comorbidities that require specific treatment [4]. Although there is strong evidence regarding the safety of CGRP mAbs in combination with “small molecule” drugs (e.g., antihypertensives, statins, antidiabetic agents, and antiseizure medications), no data are available about the safety of the combination of CGRP mAbs with other mAbs for comorbid diseases, such as psoriasis, multiple sclerosis, and autoimmune and neoplastic diseases. The combination of mAbs is recognized as a safe and successful treatment in clinical oncology, where specific combinations aim to target different pathways simultaneously, resulting in additive or synergistic effects [5].

In principle, monoclonal antibodies are suitable for use in combination due to their limited overlapping toxicity and lack of pharmacokinetic interactions [6]. However, the final direct or indirect effects of combining mAbs depend on the specific antibodies, the clinical indication, and the target population. To date, no prospective data involving patients with migraine treated with anti‐CGRP mAbs and other mAbs for comorbid diseases are available, except for a small case series of patients with multiple sclerosis (MS) treated with mAbs [7]. Only two retrospective studies, one including 27 patients with MS [8] and the other 23 patients with other neurologic, oncologic, or autoimmune conditions [9], are available.

Herein, in a multicentric, prospective observational study, we evaluated the long‐term effectiveness and tolerability of CGRP mAbs combined with other mAbs (not anti‐CGRP mAbs).

## METHODS

### Study design and ethics

In 2019, the Italian Headache Registry (*Registro Italiano per le Cefalee* [RICe]) was established to evaluate primary headache disorders in Italy. The study received approval in March 2019 with subsequent amendments. Currently, the RICe involves 60 Italian headache centers at all levels of care, and all patients attending these centers are invited to enroll. Consenting patients included in the study are followed up at each visit according to clinical practice.

We conducted an investigator‐initiated, prospective, multicenter cohort study involving patients treated with two different mAbs (an anti‐CGRP mAb and another mAb that is not an anti‐CGRP mAb) at 15 Italian headache centers. All consecutive outpatients who received at least one coadministration of both mAbs between July 2020 and June 2023 were enrolled and provided informed consent to participate in the RICe study. The study consisted of a 6‐month potential cotreatment period, starting with a 1‐month baseline (run‐in) period (for anti‐CGRP mAbs) predetermined in the study protocol but according to clinical practice. If the patient first started a non‐anti‐CGRP mAb, the baseline of the anti‐CGRP was overlapping. Patients were then assessed for effectiveness and safety based on a follow‐up of 3 or 6 months. If a patient discontinued one of the coadministered mAbs before the 6‐month follow‐up, the cause of discontinuation and adverse events (AEs; if applicable) were reported.

The study was reported according to the STROBE (Strengthening the Reporting of Observational Studies in Epidemiology) guidelines and is a part of the RICe study, approved by the local ethics committee of *Careggi* University Hospital (CEAVC Studio RICe, 14591_oss and subsequent amendments 2022‐609).

### Patient features and variables collected

The study recruited adult patients diagnosed with high‐frequency episodic migraine (8–14 migraine headache days/month) or chronic migraine (CM) according to the International Classification of Headache Disorders 3rd Edition criteria [10], with or without medication overuse (MO), who initiated an anti‐CGRP mAb and an mAb for another disease (other mAb). For patients with MO, prior detoxification strategies were not mandatory. To access anti‐CGRP mAbs, in accordance with Italian regulations, patients were required to have reported previous failures with at least three preventive treatments, which could include tricyclic antidepressants, beta‐blockers, antiseizure medications, and onabotulinumtoxinA (only for CM), due to lack of either efficacy or tolerability, together with ≥8 monthly days of debilitating headache and a Migraine Disability Assessment (MIDAS) score of ≥11.

Throughout the cotreatment phase and during baseline (for anti‐CGRP mAbs), patients maintained a headache diary, recording their monthly headache days (MHDs) and acute medication use, including both the absolute number of analgesics (AMNs) and the number of days with at least one analgesic used (AMDs) per month. A headache day was defined as any day a patient experienced any type of headache. Response rates were evaluated based on reductions in MHDs of 30%, 50%, 75%, or 100%. In this study, a month was defined as 30 days.

Additionally, patients completed the Headache Impact Test‐6 (HIT‐6) questionnaire monthly and the MIDAS questionnaire quarterly. Both questionnaires are widely used and validated in migraine research [11, 12]. The Patients' Global Impression of Change (PGIC) scale (from 0 [no change or worsening] to 10 [a great and decisive improvement]) was employed to assess patients' impressions of the effectiveness of both treatments (anti‐CGRP and other mAbs) [13]. Any AEs experienced during the treatment were reported. Demographic information, migraine characteristics (including the presence of aura, disease duration, and onset of migraine), prior treatment failures with various drug classes (such as beta‐blockers, tricyclic antidepressants, antiseizure medications, and onabotulinumtoxinA), and current preventive and acute symptomatic treatments were collected. Patients were allowed to use other preventive medications as per clinical practice if stable for at least 3 months before anti‐CGRP mAbs baseline.

### Outcomes and analysis

The primary outcomes included the percentage of patients who discontinued one or both of the mAbs due to AEs, with particular consideration of AEs different from those usually reported in monotherapy. A board of three clinicians (L.F.I., F.P., Gennaro Saporito) was established to assess the reported AEs and the reason for treatment discontinuation and to discuss their causal relationship with one of the target treatments.

The secondary effectiveness outcomes included the absolute change from baseline in MHDs, response rates (≥30%, ≥50% ≥75%, and 100% reduction in MHDs), the persistence in MO at months 3 and 6 compared to baseline, and the description of PGIC scores for both mAbs (as ordinal variable [0–10] or categorized ≥5). Furthermore, the absolute changes from baseline in the AMDs and AMNs, as well as scores on the MIDAS and HIT‐6 questionnaires, were evaluated at the same follow‐ups. All patients with at least 6 months of follow‐up were included in the study, regardless of treatment discontinuation due to AEs, ineffectiveness, or loss to follow‐up. All outcome time frames refer to the indicated month. All analyses were conducted on the overall population.

### Statistical analysis and missing data

Continuous variables are reported as mean ± SD or median (interquartile range) and categorical data as number (percentage). The normality assumption was assessed using the Shapiro–Wilk test, and because the data were not normally distributed, a Wilcoxon signed‐rank test was performed to evaluate pre–post changes in quantitative variables and an exact McNemar test for categorical dependent variables. The study's sample size was not determined based on statistical considerations; all consecutive outpatients were included. Because few patients had missing data for the variables, no imputation was performed for missing data, and the number of patients analyzed is reported in figures and table legends, as appropriate.

For all variables, a significance level of two‐tailed *p* < 0.05 was considered statistically significant, and the Bonferroni correction was used for multiple comparisons. SPSS software version 26.0 (IBM, Armonk, NY, USA) was used for all data analyses, and Prism version 9.00 (GraphPad, La Jolla, CA, USA) was used to create the graphs.

## RESULTS

### Baseline characteristics

Thirty‐eight patients (32 women [84.2%], mean age ± SD = 50.7 ± 10.0) were included. Table 1 and Table S1 provide further details on the demographic and clinical features. In most cases, (*n* = 27/38, 71.1%), anti‐CGRP mAb (erenumab [23.7%], galcanezumab [39.5%], or fremanezumab [7.9%]) was added to a previously ongoing treatment with another mAb (namely adalimumab, alirocumab, belimumab, benralizumab, certolizumab, denosumab, dupilumab, etanercept, golimumab, ocrelizumab, omalizumab, natalizumab, ocrelizumab, omalizumab, risankizumab, tocilizumab, or ustekinumab; Table 2 and Table S2). The most common diseases associated with migraine were osteoporosis (*n* = 9, 23.7%), psoriatic arthritis (*n* = 6, 15.8%), and rheumatoid arthritis (*n* = 6, 15.8%), followed by ankylosing spondylitis (*n* = 4, 10.5%) and MS (*n* = 4, 10.5%). Other diseases included asthma (*n* = 2, 5.3%), ulcerative colitis, Crohn disease, vasculitis, Behcet disease, systemic lupus erythematosus, and dyslipidemia and chronic rhinosinusitis with one case each (*n* = 1, 2.6%).

**TABLE 1:** Patient demographic and clinical features at baseline.

Feature	Value
Overall population, *N*	38
**Demographics**	
Age, years, mean ± SD	50.7 ± 10.0
Sex female, *n* (%)	32 (84.2)
**Migraine features**	
Chronic migraine, *n* (%)	30 (78.9)
Medication overuse, *n* (%)	26 (68.4)
Prior preventive ineffective drugs, mean ± SD	3.7 ± 1.2
Monthly headache days, mean ± SD	20.6 ± 7.1
Days with at least one analgesic use, mean ± SD	18.2 ± 7.7
Absolute number of analgesics, mean (95% CI)	23.7 (18.1–29.4)
MIDAS score, mean ± SD	79.7 ± 55.2
HIT‐6 score, mean ± SD	66.6 ± 9.5

*Note*: Percentages are of total patients.

Abbreviations: CI, confidence interval; HIT‐6, Headache Impact Test‐6; MIDAS, Migraine Disability Assessment; SD, standard deviation.

**TABLE 2:** Monoclonal antibodies coadministered.

	*n* (% of overall population)
Patients	38 (100)
**Anti‐CGRP mAbs**	
Erenumab	12 (31.6)
Galcanezumab	18 (47.4)
Fremanezumab	8 (21.1)
**Other mAbs in combination**	
TNF‐alpha inhibitors	
Adalimumab	9 (23.7)
Certolizumab	3 (7.9)
Etanercept	2 (5.3)
Golimumab	1 (2.6)
B‐cell depletion	
Ocrelizumab	2 (5.3)
Belimumab	1 (2.6)
Others	
Omalizumab	2 (5.3)
Natalizumab	2 (5.3)
Ustekinumab	1 (2.6)
Risankizumab	1 (2.6)
Tocilizumab	2 (5.4)
Denosumab	9 (23.7)
Alirocumab	1 (2.6)
Benralizumab	1 (2.6)
Dupilumab	1 (2.6)

*Note*: Percentages are of total patients.

Abbreviations: CGRP, calcitonin gene‐related peptide; mAb, monoclonal antibody; TNF, tumor necrosis factor.

### mAb discontinuation

**FIGURE 1:**
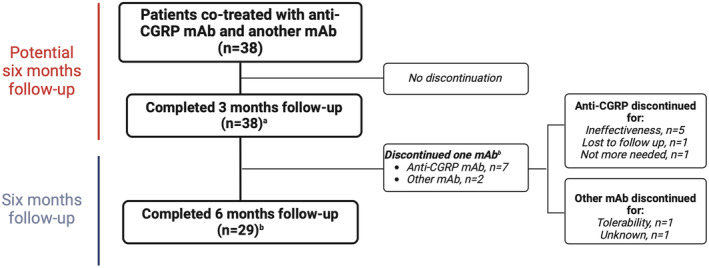
Flowchart of patients. All values in the flowchart represent the number of patients if not otherwise specified. ^a^Patients with coadministration. ^b^One patient discontinued both monoclonal antibodies (mAbs). CGRP, calcitonin gene‐related peptide.

Nine patients (23.6%) discontinued one of the two mAbs before the end of treatment (seven patients discontinued the anti‐CGRP mAb and two patients the other mAb). Anti‐CGRP mAbs were discontinued mainly due to ineffectiveness (*n* = 5/9, 55.5%), one for clinical remission (i.e., anti‐CGRP mAb no longer needed; *n* = 1/9, 11.1%), and one for lost to follow‐up (*n* = 1/9, 11.1%). All reasons for discontinuation are reported in Table S3. A flowchart of patients is presented in Figure 1.

### Safety and tolerability

Mild AEs associated with the starting of the second mAb were detected in six patients (15.8%), with three cases of constipation (7.9%), one case of alopecia, and one case of combined gastrointestinal symptoms (i.e., nausea, vomiting, diarrhea, and abdominal pain; 2.6% each). In only the latter case, AEs led to the discontinuation of treatment with the second mAb (alirocumab) in combination with erenumab.

In the three cases of constipation, the onset was temporally associated with the introduction of a CGRP mAbs (erenumab, *n* = 1 or galcanezumab, *n* = 2) to a pre‐existing mAbs therapy (denosumab, ocrelizumab, natalizumab), and in one case with the addition of adalimumab to fremanezumab. These AEs are not related to direct or indirect pharmacokinetics interactions.

### Anti‐CGRP effectiveness analysis

All patients treated with anti‐CGRP mAbs completed 3 months of treatment, and 31 (81.6%) completed 6 months of treatment, regardless of the other mAb. Considering only the coadministration effectiveness, all patients completed 3 months of coadministration (*n* = 38), with 29 patients (76.3%) completing 6 months of cotreatment (Figure 1).

**FIGURE 2:**
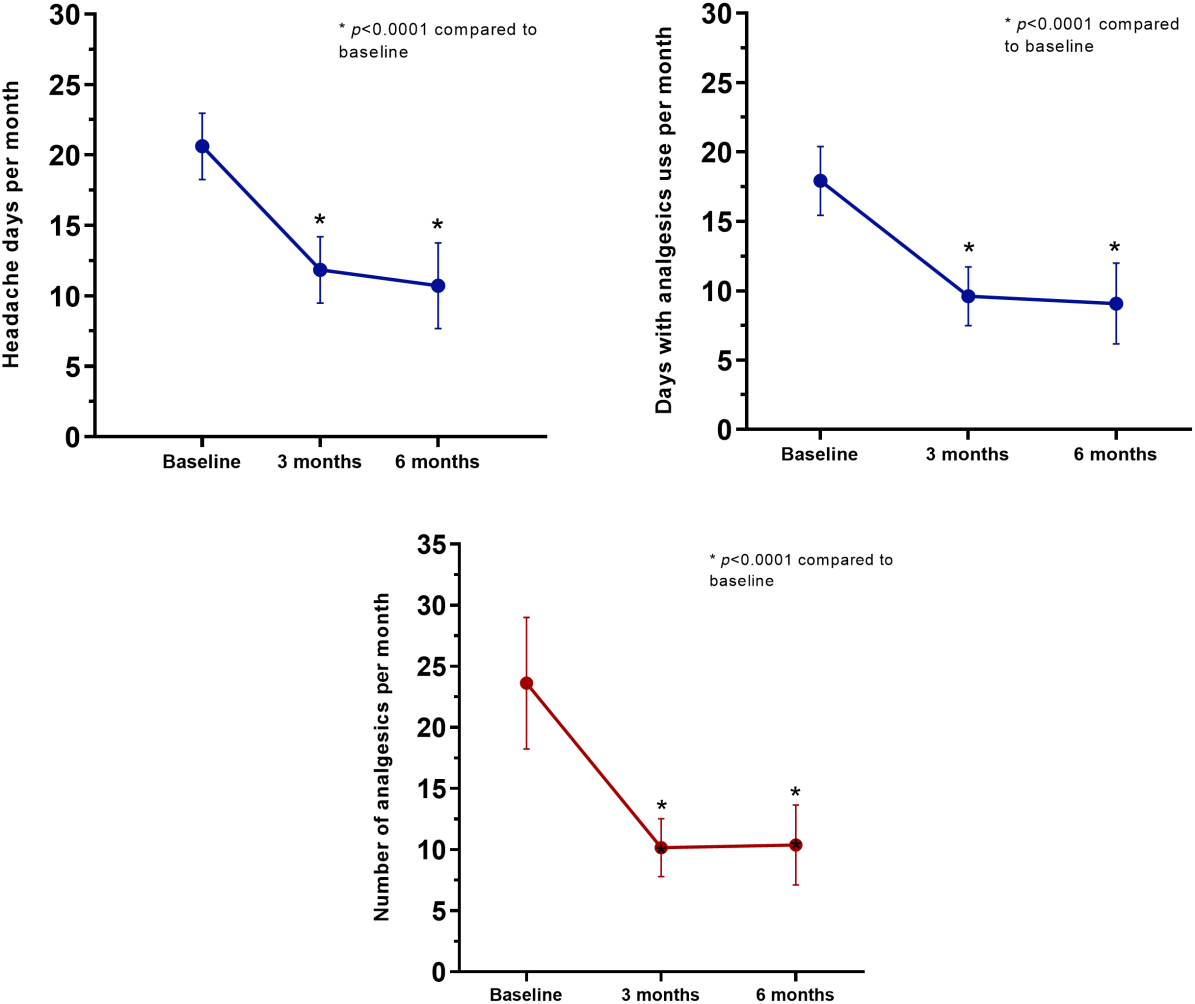
Number of monthly headache days and days with analgesics use per month at baseline, 3 months, and 6 months of treatment with calcitonin gene‐related peptide monoclonal antibodies in combination with another monoclonal antibody. Error bars represent 95% confidence interval.

MHDs significantly decreased from baseline to 3 months (*z* = −4.7, *p* < 0.001) and 6 months (*z* = −4.1, *p* < 0.001), as did the MIDAS score (*z* = −4.8 at 3 months and *z* = −4.1 at 6 months, *p* < 0.001) and the HIT‐6 score (*z* = −4.0 at 3 months and *z* = −3.7 at 6 months, *p* < 0.001; Figure 2 and Table 2). Regarding symptomatic drug use, there was a decrease in both AMDs (*z* = −4.5 at 3 months and *z* = −3.7 at 6 months, *p* < 0.001) and AMNs (*z* = −4.8 at 3 months and *z* = −3.8 at 6 months, *p* < 0.001). No significant differences were found between month 3 and month 6 in MHDs, MIDAS, and symptomatic drug use (AMNs and AMDs), whereas HIT‐6 had a significant decrease (*z* = −2.0, *p* = 0.03). Data are summarized in Table 3, Figure 2, and Figure S1.

**TABLE 3:** Changes in migraine‐related variables during cotreatment with anti‐calcitonin gene‐related peptide mAb and another mAb.

	Baseline, *n* = 38	3 months, *n* = 38	6 months, *n* = 29
Monthly headache days	21.0 (12.0)	−8.0 (12.5)	−9.0 (16.0)
Days with at least one analgesic, *n*	18.0 (14.0)	−8.0 (10.0)	−8.5 (13.2)
Absolute number of analgesics	20.0 (19.0)	−9.0 (16.0)	−10.0 (19.5)
MIDAS score	61.5 (71.0)	−37.5 (63.7)	−52.5 (67.5)
HIT‐6 score	69.0 (11.0)	−7.0 (14.5)	−15.5 (14.5)

*Note*: Values are reported as median (IQR).

Abbreviations: HIT‐6, Headache Impact Test‐6; mAb, monoclonal antibody; MIDAS, Migraine Disability Assessment.

**FIGURE 3:**
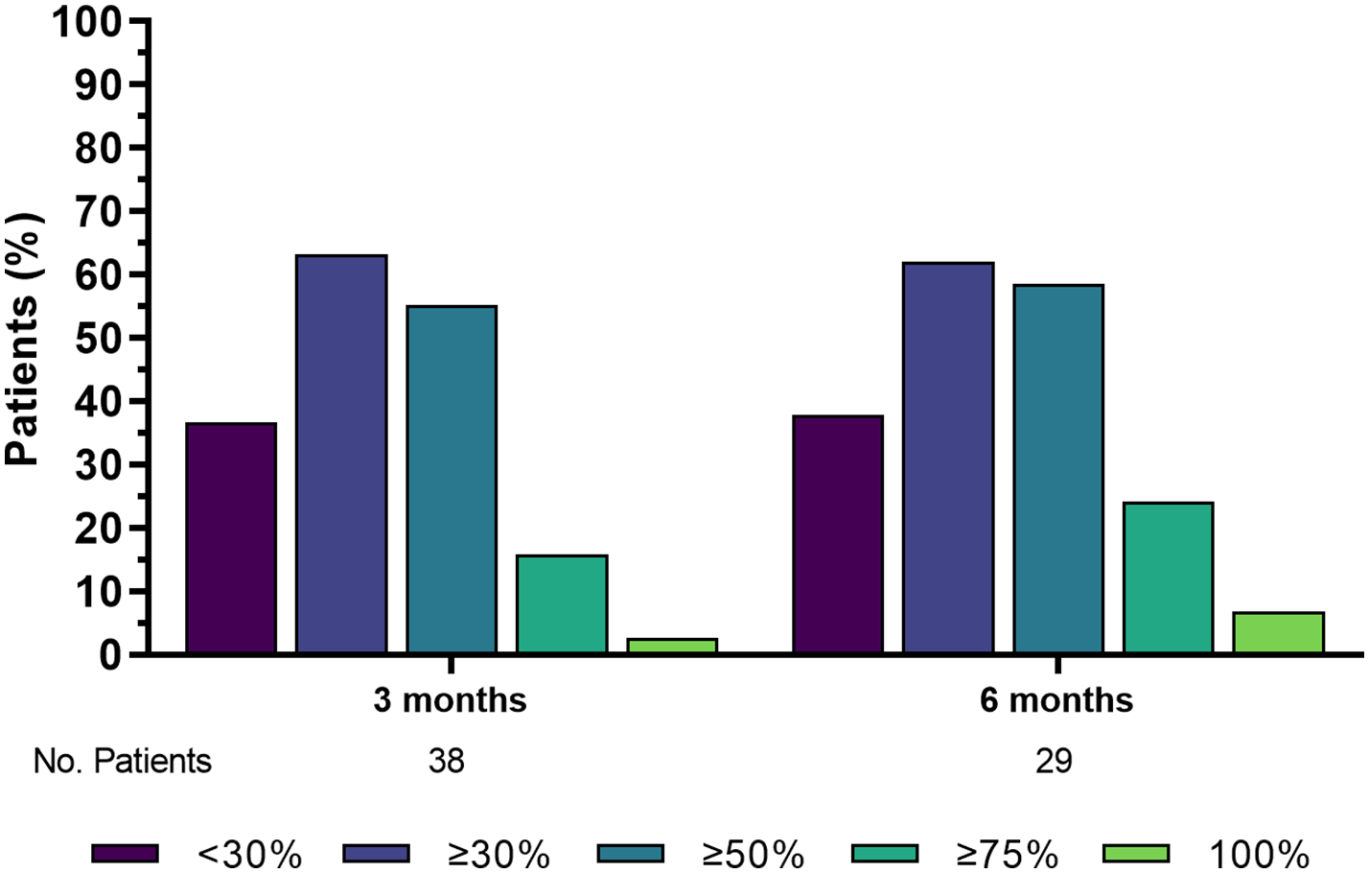
Response rates in the population with coadministration of monoclonal antibodies. Percentages are calculated of the total number of patients per follow‐up as reported in Table S3.

At month 3 (*n* = 38), 24 (63.2%) patients achieved a ≥30%, 21 (55.3%) a ≥50%, and six (15.8%) a ≥75% cumulative response rate. One patient achieved a 100% reduction in MHDs. At month 6 of follow‐up (*n* = 29), 18 (62.0%) achieved a ≥30% reduction in MHDs, 17 (58.6%) a ≥50% reduction, and seven (24.1%) a ≥75% cumulative response rate. Two patients (6.9%) achieved a 100% reduction in MHDs (Figure 3 and Table S4).

The overall clinical benefit expressed with the PGIC score for anti‐CGRP mAbs was 4.7 ± 2.8 and 5.2 ± 3.0 at 3 and 6 months, respectively. Categorizing PGIC scores (using the cutoff of ≥5), 27.4% of patients (18/38) reported a PGIC ≥ 5 at 3 months and 48.3% (14/29) at 6 months. For the other mAb, the PGIC score was 4.6 ± 2.9 and 4.6 ± 3.1 at 3 and 6 months, respectively. Categorizing PGIC scores, 47.4% of patients (18/38) reported a PGIC ≥ 5 at 3 months and 41.4% (12/29) at 6 months.

## DISCUSSION

Over the past decades, mAb therapies have revolutionized the management of several disorders, including cancer and rheumatologic and immune‐related disorders, as well as MS and migraine. Recently, they are emerging as a major class of therapeutics for infectious diseases. The number of approved and marketed mAbs reached 162 in June 2022, but the number of authorized therapies is continuously evolving, with new treatments constantly being developed and approved [14]. Therefore, with the increasing use of anti‐CGRP mAbs for migraine and the increasing number of mAbs marketed, it is likely that a patient starting a CGRP mAb is already receiving or will receive another mAb for a different concomitant disease.

To date, scarce data about the safety of the combination of CGRP mAbs with other mAbs are available. A small case series of nine patients showed that the use of anti‐CGRP mAbs for migraine in people with relapsing–remitting MS was associated with AEs in 33% of the cases, with two patients experiencing, respectively, a progression in the Expanded Disability Status Scale score and a new radiological relapse [7].

In a retrospective case series including 27 patients with MS, concurrent use of disease‐modifying therapy and anti‐CGRP mAbs was not associated with the worsening of MS symptoms, and only mild AEs occurred in 11% of patients (i.e., muscle spasms and constipation) [8].

Another retrospective case series included 23 patients with migraine treated with an anti‐CGRP mAb and comorbid neurologic, oncologic, or autoimmune diseases treated with another mAb. No evidence of novel AEs with coadministration of mAbs was reported. The reported AEs found during the study were constipation, hair loss, and injection site reaction, all common AEs occurring with anti‐CGRP mAbs [9]. Furthermore, the effectiveness of anti‐CGRP mAbs in patients with rheumatologic or autoimmune comorbidities has not been thoroughly investigated. Limited data suggest that the presence of immunorheumatologic disease history may be a negative predictive factor for the responsiveness of migraine patients to anti‐CGRP mAbs [1, 15].

In our population, most of the patients were already being treated with a mAbs for another condition, mainly for osteoporosis, rheumatologic diseases, or MS. These disorders often occur in comorbidity with migraine [16], and the mAbs used in these conditions have been available for a longer time compared to CGRP mAbs. The incidence of AEs following the introduction of a second mAb was very low and mostly mild. AEs associated with the introduction of the second mAb included constipation, other gastrointestinal symptoms, and alopecia. The AEs were usually reported during anti‐CGRP mAb treatment in monotherapy. In most cases, the onset of constipation was temporally associated with the introduction of an anti‐CGRP mAb (erenumab or galcanezumab) to a pre‐existing mAb therapy (denosumab, ocrelizumab, natalizumab). In the case of erenumab, it is reasonable to attribute the onset of constipation to erenumab itself, as constipation is more frequent in erenumab‐treated patients compared to patients managed with other CGRP mAbs [17, 18].

Regarding galcanezumab, our data indicate a temporal association between its introduction and the onset of constipation when combined with ongoing prior therapies such as natalizumab or ocrelizumab. Alopecia was reported following the combination of galcanezumab with previously prescribed ustekinumab. Both galcanezumab and ustekinumab have been reported to cause hair loss individually [19, 20]. In both cases, it is challenging to determine which drug determined the onset or worsening of gastrointestinal symptoms, as well as to determine causation or correlation.

mAbs substantially differ from small molecules in pharmacodynamic and pharmacokinetic features. The target specificity of mAbs is associated with fewer AEs compared to conventional small molecules, with occurring AEs usually strictly related to the target. Considering pharmacokinetics, mAbs are considerably larger than most small molecules, distribution across the blood–brain barrier is poor, and they have a different clearance mechanism with small molecules (i.e., catabolism to endogenous amino acids) and no implication of cytochrome CYP450‐mediated metabolism. Therefore, interaction between mAbs and between small molecules and mAbs is unlikely, and if these occur, they are mostly through indirect mechanisms [6, 21]. Differences between small molecules and mAbs have been reported [6, 22]. To date, no interactions have been reported with anti‐CGRP mAbs and other molecules [6]. Providing real‐world evidence on the absence of interactions between anti‐CGRP mAbs and other mAbs can boost the confidence of specialists in co‐prescribing mAbs in clinical practice.

This study has several strengths. It is the first and largest multicentric study with data collected prospectively, specifically assessing the safety and tolerability of coadministration of anti‐CGRP mAbs and other mAbs. Several different mAbs and diseases have been included, allowing a widespread overview of clinical practice. There are also some limitations to acknowledge. First, due to data availability within clinical practice settings, MHDs were used as secondary effectiveness outcomes instead of monthly migraine days. Second, it is difficult to determine whether an AE can conclusively be attributed to either the anti‐CGRP mAb or another mAb.

## CONCLUSIONS

Preliminary findings confirm the efficacy and safety of anti‐CGRP mAbs even when used in combination with other mAbs for other diseases. The appearance of mild AEs following the start of combined therapies should be further investigated to determine whether there is a temporal or causal relationship between the two events.

## AUTHOR CONTRIBUTIONS

**Luigi Francesco Iannone:** Conceptualization; investigation; writing – original draft; software; formal analysis; project administration; methodology; visualization; validation; writing – review and editing; data curation; supervision. **Marina Romozzi:** Data curation; writing – review and editing; writing – original draft; formal analysis. **Antonio Russo:** Conceptualization; investigation; visualization. **Gennaro Saporito:** Investigation. **Federico De Santis:** Investigation. **Raffaele Ornello:** Investigation. **Grazia Sances:** Investigation. **Gloria Vaghi:** Investigation; writing – review and editing. **Cristina Tassorelli:** Investigation. **Maria Albanese:** Investigation. **Simona Guerzoni:** Investigation. **Alfonsina Casalena:** Investigation. **Catello Vollono:** Investigation. **Paolo Calabresi:** Investigation. **Maria Pia Prudenzano:** Investigation. **Edoardo Mampreso:** Investigation. **Giorgio Dalla Volta:** Investigation. **Maria Rosaria Valente:** Investigation. **Gianluca Avino:** Investigation. **Alberto Chiarugi:** Investigation. **Simona Sacco:** Investigation. **Francesca Pistoia:** Investigation; validation; methodology; writing – review and editing; writing – original draft; conceptualization; visualization; formal analysis; project administration; data curation; supervision.

## CONFLICT OF INTEREST STATEMENT

L.F.I. has received fees and honoraria for advisory boards or speaker panels from Teva, Eli Lilly, Lundbeck, Pfizer, and AbbVie. G.Sap. has received fees and honoraria for advisory boards or speaker panels from Eli Lilly, Lundbeck, Pfizer, and Teva. G.V. has received personal fees from Lundbeck. M.A. has received personal fees from Eli Lilly, Lundbeck, Teva, Pfizer, and AbbVie/Allergan. P.C. has received research support, speaker honoraria, and support to attend national and international conferences from AbbVie, Bayer Schering, Bial, Biogen‐Dompè, Biogen‐Idec, Eisai, Genzyme, Lundbeck, Lusofarmaco, Merck‐Serono, Novartis, Prexton, Teva, UCB Pharma, and Zambon. R.O. reports personal fees and nonfinancial support from Allergan‐AbbVie, Eli Lilly, Novartis, Pfizer, and Teva. S.G. has received fees and honoraria for advisory boards, speaker panels, or clinical investigation studies from Novartis, Teva, Eli Lilly, Pfizer, Lundbeck, Angelini, and AbbVie. S.S. reports personal fees from Abbott, Allergan‐AbbVie, AstraZeneca, Boehringer, Eli Lilly, Lundbeck, Novartis, Novo Nordisk, Pfizer, and Teva and research grants from Novartis and Uriach. The other authors have no conflicting interests.

## Supporting information


**DATA S1** Migraine Disability Assessment (MIDAS) and Headache Impact Test‐6 (HIT‐6) scores at baseline, 3 months, and 6 months of treatment with calcitonin gene‐related peptide monoclonal antibodies in combination with another monoclonal antibody.

## Data Availability

Data supporting the findings in the present study are reported in the article and in the supplementary materials. The raw data collected and analyzed are available from the corresponding author on reasonable request.
