# Supplementary material for: Association of anti‐calcitonin gene‐related peptide with other monoclonal antibodies for different diseases: A multicenter, prospective, cohort study
Source: Eur J Neurol. 2024 Sep 16;31(12):e16450. doi: 10.1111/ene.16450 (PMC11555159; doi:10.1111/ene.16450)
Supplement: Supplementary file 1 — DATA S1 Migraine Disability Assessment (MIDAS) and Headache Impact Test‐6 (HIT‐6) scores at baseline, 3 months, and 6 months of treatment with calcitonin gene‐related peptide monoclonal antibodies in combination with another monoclonal antibody. [file ENE-31-e16450-s001.docx]

**Association of anti-CGRP with other monoclonal antibodies for different diseases: a multicenter, prospective, cohort study**

*Supplementary data*

| **Table S1.** Patients demographic and clinical features at baseline, month 3 and month 6 (median, IQR). | |
| --- | --- |
|  | **Median (IQR)** |
| Age [years] | 51.5 (12) |
| Prior preventive ineffective drugs | 4.0 (2) |
| ***Baseline,* n =38** |  |
| Monthly headache days | 21.0 (12) |
| Days with at least one analgesic use | 18 (14) |
| Analgesics number | 20.0 (19) |
| MIDAS score | 61.5 (71) |
| HIT-6 score | 69.0 (11) |
| ***Month-3,* n =38** |  |
| Monthly headache days | 10.0 (8) |
| Days with at least one analgesic use | 8.0 (8) |
| Analgesics number | 8.5 (8) |
| MIDAS score | 28.0 (35) |
| HIT-6 score | 59.0 (12) |
| ***Month-6,* n =29** |  |
| Monthly headache days | 8.0 (9) |
| Days with at least one analgesic use | 8.0 (6) |
| Analgesics number | 8.0 (10) |
| MIDAS score | 18.0 (36) |
| HIT-6 score | 56.0 (12) |
| *CGRP, calcitonin gene related peptide; HIT-6, Headache Impact Test 6; mAb, monoclonal antibody; MIDAS, Migraine Disability Assessment.*  *At baseline, month-3 and month-6: HIT-6 score is calculated on 32, 31 and 23 patients, MIDAS score on 36, 36 and 27 patients, AMDs on 37,36 and 26 patients, AMNs on 37, 36 and 26 patients, respectively.* | |

|  | **[Table S2.](#_bookmark14)** Details on coadministration treatments (n=38). | | | | | | | | | | | | | | | |
| --- | --- | --- | --- | --- | --- | --- | --- | --- | --- | --- | --- | --- | --- | --- | --- | --- |
|  | | **ALI** | **ADA** | **BEL** | **BEN** | **CRT** | **DEN** | **DUP** | **ETN** | **GOL** | **NAT** | **OMA** | **OCR** | **RIC** | **TOC** | **UST** |
| **ERE** | | 1 | 2 | - | 1 | 1 | 2 | - | - | - | 1 | - | 1 | 1 | 2 | - |
| **FRE** | | - | 2 | 1 | - | - | 3 | 1 | - | - | - | 1 | - | - | - | - |
| **GLC** | | - | 5 | - | - | 2 | 4 | - | 2 | 1 | 1 | 1 | 1 | - | - | 1 |
| **All** | | **1** | **9** | **1** | **1** | **3** | **9** | **1** | **2** | **1** | **2** | **2** | **2** | **1** | **2** | **1** |
|  | *Expressed as number (percentage), independently if first or second introduced mAb. ALI, Alirocumab; ADA, Adalimumab; BEL, Belimumab; BEN, Benralizumab; CRT, Certolizumab; DEN, Denosumab; DUP, Dupilumab; ERE, erenumab; ETN, Etanercept; FRE, Fremanezumab; GLC, Galcanezumab; GOL, Golimumab; NAT, Natalizumab; OMA, Omalizumab; OCR, Ocrelizumab; RIS, Risankizumab; TOC, Tocilizumab; UST, Ustekinumab* | | | | | | | | | | | | | | | |

| **Table S3.** Reasons for monoclonal antibodies discontinuation. | |
| --- | --- |
|  | **Discontinuation**  **(n=9)** |
| ***Anti-CGRP mAbs*** |  |
| Ineffectiveness | 5 (55.5) |
| Clinical remission | 1 (11.1) |
| Lost to follow-up | 1 (11.1) |
| ***Other mAbs*** |  |
| Adverse events | 1 (11.1) |
| Reason not provided | 1 (11.1) |
| *Percentages are expressed on column total. CGRP, calcitonin gene related peptide; mAb, monoclonal antibody.* | |

| **Table S4.** Response rate during co-treatment (observed data analysis). | | |
| --- | --- | --- |
|  | **3 months** | **6 months** |
| **Overall population**, n (%) | **(n=38)** | **(n=29)** |
| Response rate ≥30% | 24 (63.2) | 28 (62.0) |
| Response rate ≥50% | 21 (55.3) | 17 (58.6) |
| Response rate ≥75% | 6 (15.8) | 7 (24.1) |
| Response rate 100% | 1 (2.6) | 2 (6.9) |
| *Percentages are expressed on column total. Response rate percentages are cumulative.* | | |


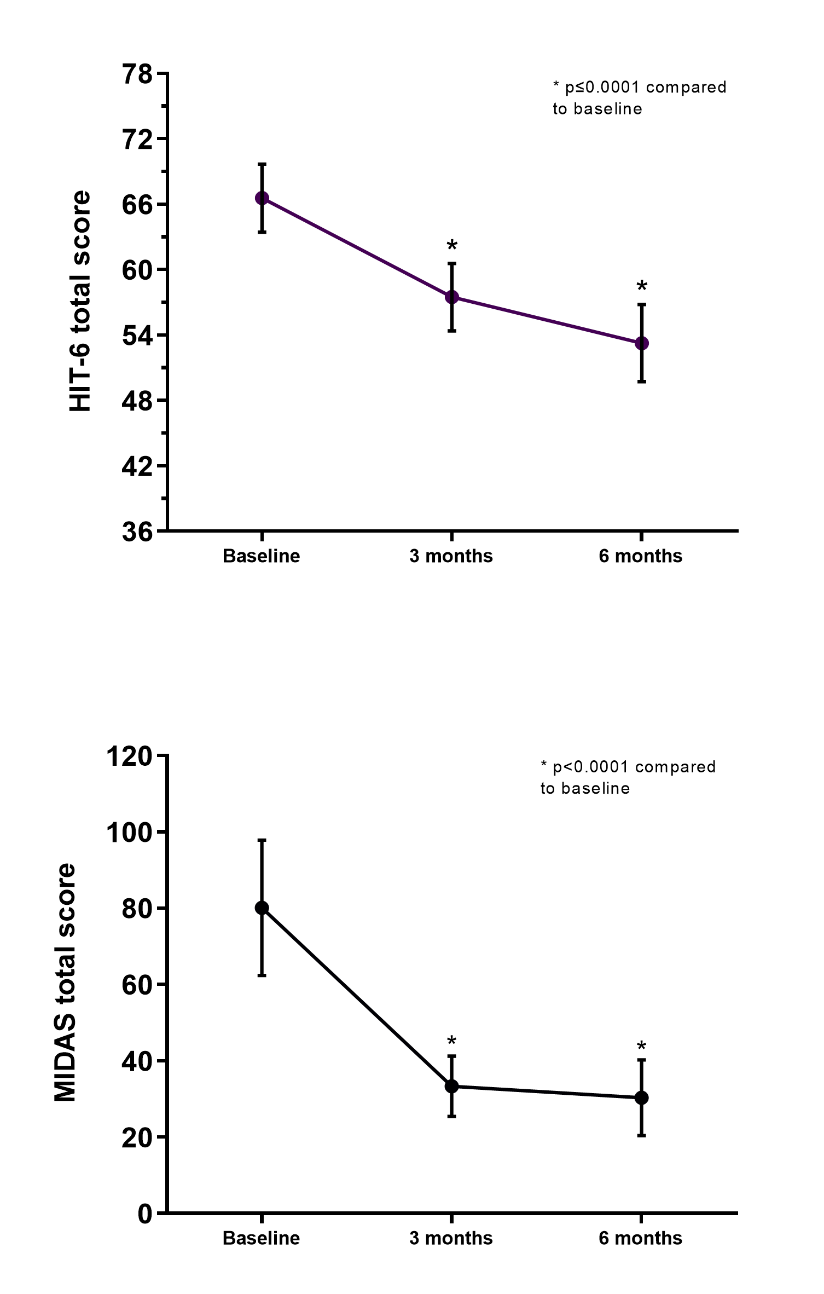


**Figure S1.** Headache Impact Test 6 (HIT-6) score, and Migraine Disability Assessment (MIDAS) score at baseline, three months, and 6 months of treatment with CGRP-mAbs in combination with another monoclonal antibody. *Error bars represent 95% CI.*
